# A Comparison of Podocyturia, Albuminuria and Nephrinuria in Predicting the Development of Preeclampsia: A Prospective Study

**DOI:** 10.1371/journal.pone.0101445

**Published:** 2014-07-10

**Authors:** Belinda Jim, Swati Mehta, Andi Qipo, Kwanghee Kim, Hillel W. Cohen, Robert M. Moore, John C. He, Shuchita Sharma

**Affiliations:** 1 Jacobi Medical Center, Albert Einstein College of Medicine, Department of Nephrology/Medicine, Bronx, New York, United States of America; 2 James J. Peters VA Medical Center, Department of Nephrology/Medicine, Bronx, New York, United States of America; 3 Providence Alaska Medical Center, Anchorage, Alaska, United States of America; 4 Albert Einstein College of Medicine, Department of Epidemiology and Population Health, Bronx, New York, United States of America; 5 Jacobi Medical Center, Albert Einstein College of Medicine, Department of Obstetrics and Gynecology, Bronx, New York, United States of America; 6 Mount Sinai School of Medicine, Department of Nephrology/Medicine, New York, New York, United States of America; Max-Delbrück Center for Molecular Medicine (MDC), Germany

## Abstract

Preeclampsia, a hypertensive multisystem disease that complicates 5–8% of all pregnancy, is a major cause for maternal and fetal mortality and morbidity. The disease is associated with increased spontaneous and evoked preterm birth and remote cardio-renal disorders in the mother and offspring. Thus the ability to predict the disease should lead to earlier care and decreased morbidity. This has led to fervent attempts to identify early predictive biomarkers and research endeavors that have expanded as we learn more regarding possible causes of the disease. As preeclampsia is associated with specific renal pathology including podocyte injury, early urinary podocyte (podocyturia), or the podocyte specific proteinuria nephrin in the urine (nephrinuria), as well as the more easily measured urinary albumin (albuminuria), have all been suggested as predictive markers. We performed a prospective study recruiting 91 pregnant women (78 of whom were high risk) and studied the predictive ability of these three urinary biomarkers. The subjects were recruited between 15–38 weeks of gestation. Fourteen patients, all in the high-risk obstetric group, developed preeclampsia. The levels of podocyturia, nephrinuria, and albuminuria were variably higher in the high-risk pregnant patients who developed preeclampsia. The sensitivities and specificities for podocyturia were 70% and 43%, for albuminuria were 36% and 96%, and for nephrinuria were 57% and 58%, respectively. Also, abnormal nephrinuria (69%) and podocyturia (38%) were detected in low risk women who had uncomplicated gestations; none of these women exhibited albuminuria. In our study, none of the three urinary markers achieved the minimum predictive values required for clinical testing. The lack of excessive albuminuria, however, may indicate a preeclampsia-free gestation. Given a discrepant literature, further studies with larger sample size should be considered.

## Introduction

Preeclampsia (PE) is a hypertensive complication of pregnancy and a major cause of maternal and fetal morbidity and mortality worldwide. The disease involves many organs and its life-threatening events include cerebral hemorrhage, hepatic failure, pulmonary edema, disseminated intravascular coagulation accounting to 10–15 percent of maternal deaths worldwide [1]. Given the ominous outcomes associated with PE, a reliable diagnostic and predictive tool could lead to early interventions and substantially decrease poor outcomes. In this respect, a variety of biomarkers have recently been identified concurrent with progress made regarding our understanding of the pathogenesis of the disease. For example, changes in circulating levels of angiogenic factors, such as placental growth factor (PlGF), soluble fms-like tyrosine kinase-1 (sFlt-1), and soluble endoglin (sEng) have been shown to occur prior to the onset of PE and cause phenotypic signs (e.g., hypertension proteinuria, glomerular endotheliosis) in animal models [2]–[7]. These angiogenic factors also appear to be specific for PE and help differentiate it from other hypertensive conditions such as acute and chronic glomerulonephritis [8], [9] and other hypertensive disorders [10]. However, the value of these angiogenic factors in predicting the development of PE in larger population is still questionable as their test characteristics are wide-ranging and not consistent [11].

Interest in using podocytes as a predictive and diagnostic marker for PE stems from the observations that podocyte-specific protein expression is reduced in renal biopsies of patients with PE [12]. The same group detected increased urinary podocyte excretion in 15 women with PE, and when stained for podocin, a podocyte-specific protein, reported 100% sensitivity and specificity for diagnosing the disease [13]. We evaluated podocyturia as a marker for diagnosing PE in a high risk obstetric population noting the test to be neither sensitive nor specific [14]. Podocyturia has also been evaluated for its predictive accuracy by Craici et al. who showed the presence of podocyturia at the end of 2^nd^ trimester to be 100% sensitive and specific in distinguishing subsequent development of PE from normotensive pregnancies, gestational hypertension, or from both groups combined [15]. Whether this finding is reproducible, especially in other high-risk pregnancies states such as diabetes and other renal disorders, is unclear. Thus we performed a prediction study comparing the levels of three different biomarkers, podocyturia, albuminuria, and nephrinuria in the second and third trimester of pregnancy, assessing the ability of each test to predict the development of PE in amongst women who are “low risk” and “high risk” for developing PE.

## Materials and Methods

We recruited both women with apparently uncomplicated gestation (“low risk”) and others at risk for pregnancy complications (“high-risk” group as described below) from the obstetric outpatient service at Jacobi Medical Center, Bronx, NY, USA. Random urine samples were obtained from the subjects in the second (weeks 15 to 26) or third trimesters (weeks 27 to 38) of gestation. Inclusion criteria for high-risk group were PE in a previous pregnancy, chronic hypertension (HTN), Type I and Type II diabetes mellitus (DM), gestational DM, chronic kidney disease (CKD), obesity (BMI>30), and systemic lupus erythematosis (SLE), all 18 years or older. Chronic HTN was defined as preexisting HTN or blood pressure of 140/90 mmHg before 20 weeks of gestation. Gestational DM was defined as any degree of glucose intolerance with onset of first recognition during pregnancy. All patients were managed until delivery and studied for the development of PE. Diagnosis of PE fulfilled the criteria of new onset of HTN with blood pressure of 140/90 mmHg after 20 weeks of gestation and proteinuria of >300 mg of protein in a 24-hour urine specimen or 1+ protein on a urinalysis sample without evidence of another cause. For superimposed PE in patients with chronic HTN or CKD, the diagnosis was made if there was a rise in blood pressure (BP) from baseline (30 mmHg systolic blood pressure or 15 mmHg diastolic BP) or if there was a 2–3 fold increase in proteinuria. This study was approved by the Internal Review Board of Albert Einstein College of Medicine.

### Urinary protein quantification

A single voided urine specimen (20 mL) was obtained immediately following recruitment. Its concentrations of albumin-to-creatinine ratio (alb/Cr) were measured by clinical laboratories at Jacobi Medical Center, Bronx, NY, USA. And elevated level of alb/Cr was defined as ≥30 mg/g. An aliquot of the urine was frozen at −80°C for urine nephrin measurement. Urinary nephrin was determined by a competitive enzyme-linked immunosorbent assay using polyclonal antibodies against the extracellular domain (amino acids 23–322) of human nephrin (Exocell Inc., Philadelphia, PA, USA). The assay was performed by Exocell Inc. according to manufacturer’s instructions. Briefly, urine samples were diluted in the range of 1∶10 to 1∶500 (depending on degree of proteinuria in the sample). A 50-µl diluted sample was added to each well already coated with rat nephrin followed by the addition of a 50-µl rabbit anti-nephrin antibody for an incubation of 60 minutes at room temperature. Plates were washed followed by the incubation with 100-µl of anti-rabbit HRP conjugate to each well for 60 minutes. After plates were washed and color developed, absorbance was read at 450 nm. An elevated level of urinary nephrin (nephrinuria) was defined as urine nephrin-to-creatinine ratio (nephrin/Cr) (mg/g) ≥0.1 mg/g. This value was based on 10 healthy controls who consistently exhibited nephrin/Cr <0.1 mg/g.

### Podocyturia measurement

Pellets containing cellular material were retrieved from an aliquot of the patient’s urine that had been centrifuged at 700 *g* for 5 min. These pellets were carefully recovered by aspirating the supernatant, washed twice with PBS and resuspended in 1 mL of PBS. Aliquots of 100 µl of the resuspended sediment were centrifuged onto slides using the Shandon Cytospin 4 Cytocentrifuge (Thermo Electron Corporation, Asheville, NC, USA), air-dried and fixed with 1∶1 acetone/methanol for 10 minutes. The slides were immersed with PBS/1% H_2_0_2_ for 15 minutes and washed with deionized water and blocked with 10% donkey serum in PBS and 2% BSA for 30 minutes. Slides were incubated overnight with monoclonal mouse anti-human synaptopodin antibody at 1∶1 dilution and rabbit polyclonal podocin antibody at 1∶500 dilution (both gifts from Dr. Peter Mundel, Massachusetts General Hospital, Boston, MA, USA). This was followed by incubation with FITC-conjugated anti-rabbit and TRITC-conjugated anti-mouse secondary antibodies at 1∶500 dilution (Life Technologies, Grand Island, NY, USA) for 30 minutes. The nuclei were counterstained with DAPI (1∶10,000 dilution). Slides were then washed and mounted with Vectashield mounting medium (Vector Laboratories, Burlingame, CA, USA). Normal kidney tissue biopsy specimen was stained in a similar manner to obtain positive control, while negative controls were carried out by incubation in the absence of the primary antibody. Staining was evaluated by using immunofluorescent microscopy (Zeiss Axioplane2 IE microscope) and the images were obtained using the Zeiss AxioCAm MRm camera (200X magnification). Images were analyzed by two investigators and podocytes were identified by co-localization of podocin and synaptopodin; these counts were expressed as number of podocytes per mg of creatinine (#pod/mg) (pod/Cr).

### Ethics

The protocol titled “Serum and Urine Biomarkers for Preeclampsia” which was approved by the Institutional Review Board of the Albert Einstein College of Medicine of Yeshiva University. This included a consent form signed by each volunteer.

### Statistical methods

GraphPad Prism version 5.02 (GraphPad Software, Inc. La Jolla, CA) and SPSS (version 20) were used for statistical analysis and for the graphs. Mann-Whitney non-parametric tests were performed to assess the differences between two given groups. Spearman correlations were calculated to assess trends for continues variables. Data were expressed as median (interquartile range) or % as appropriate. A two-tailed P value of <0.05 was considered to be statistically significant.

## Results

### Clinical variables

A total of 78 high risk obstetric patients as well as 13 women with low risk gestations were recruited. Clinical characteristics of patients presented according to the outcome of PE are seen in Table 1. Of the 78 high risk patients, 38 were recruited during the 2^nd^ trimester, and 40 during the 3^rd^ trimester, the distribution among women with low risk pregnancies were 7 recruited during the second and 6 in the third trimester respectively; each pregnancy was evaluated until delivery. The high risk women consisted of 15 women considered to be obese, 12 with Type I or II DM, 24 with gestational DM, 26 chronic hypertensives, 2 with SLE, and 1 patient with CKD. Urine alb/Cr and nephrin/Cr were measured in all volunteers, while only 72 of the 78 high risk patients had a urine pod/Cr determination. Fourteen high-risk women developed PE, while none of the low risk patients did. Among those who later had PE, the urine samples were collected an average of 13.8 weeks before onset of PE in the 2^nd^ trimester, and 6.8 weeks before onset of PE in the 3^rd^ trimester, with an average of 10.0 weeks of both trimesters combined.

**Table 1:** Patient Characteristics According to Preeclampsia Outcome.

Patient Characteristic	High Risk Patients withPreeclampsia	High Risk Patient withoutPreeclampsia	P value
	n = 14	n = 64	
Maternal Age	31.0 (27.0–36.0)	31.5 (26.3–36.8)	p = 0.98
Gestational Age at Recruitment	24.5 (18.8–32.5)	27.5 (21.8–33.00)	p = 0.29
Gestational age at delivery	36.0 (31.8–37.2)	38.4 (37.3–39.1)	p<0.0001
SBP at time of recruitment	129.0 (121.8–146.0)	119.0 (111.0–127.8)	p = 0.0002
DBP at time of recruitment	70.5 (64.8–78.3)	67.0 (60.0–71.0)	p = 0.11
1SBP at time of delivery	151.0 (143.8–164.8)	126.0 (116.0–138.0)	p<0.0001
2DBP at time of delivery	86.0 (81.5–101.5)	75.0 (65.0–84.0)	p = 0.0002
3BUN (mg/dl)	7.5 (6.00–10.5)	8.0 (6.50–10.00)	p = 0.84
Serum creatinine (mg/dl)	0.5 (0.40–0.62)	0.5 (0.50–0.70)	p = 0.59

Data median (interquartile range) or **(#/%)**.

1 SBP: systolic blood pressure.

2 DBP: diastolic blood pressure.

3 BUN: blood urea nitrogen.

### Urinary podocyte-to-creatinine ratio

**Figure 1:**
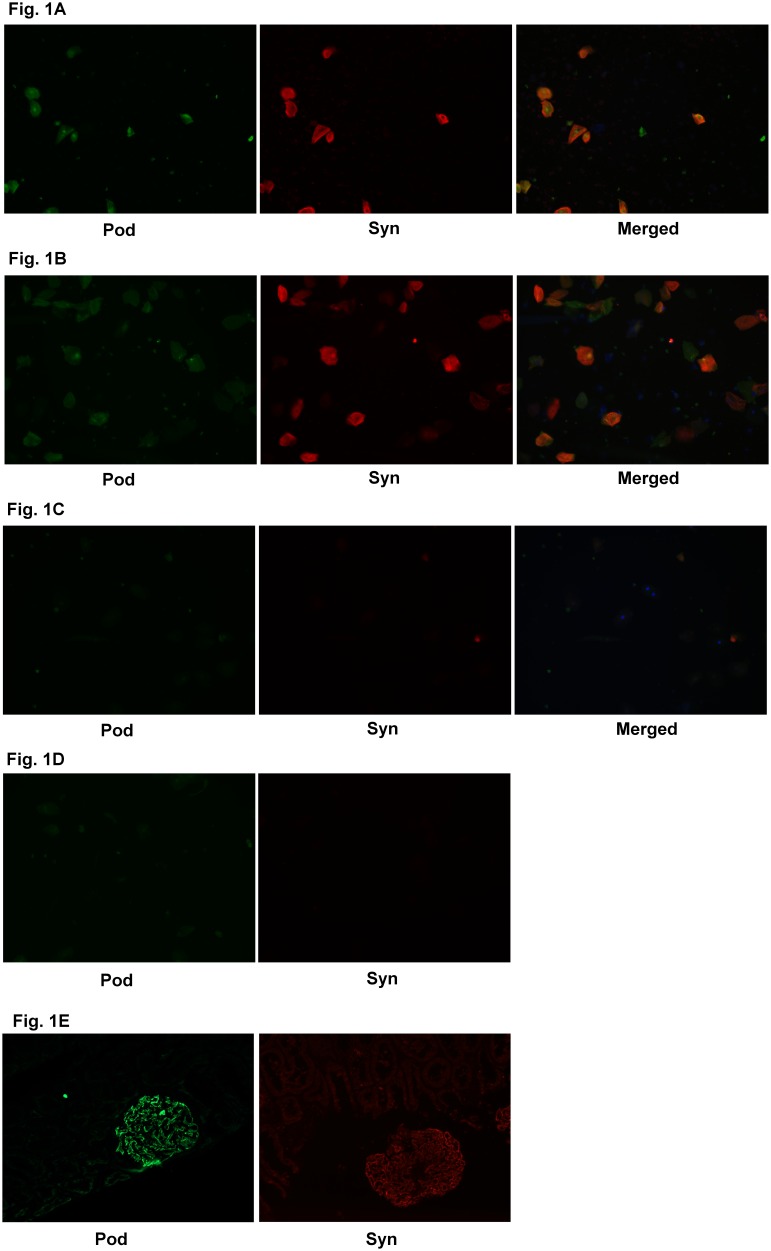
Images of Urinary Podocytes. A) Representative immunofluorescent images of urinary podocytes in high risk patients with PE stained with podocin (pod), synaptopodin (syn) and colocalized (merged). B) Representative immunofluorescent images of urinary podocytes in high risk patients without PE stained with podocin (pod), synaptopodin (syn) and colocalized (merged). C): Representative immunofluorescent images of urinary podocytes in healthy pregnant control patients stained with podocin (pod), synaptopodin (syn) and colocalized (merged). D): Negative control in absence of primary antibody E) Positive control of podocin (pod) and synaptodin (syn) on normal kidney tissue.

**Figure 2:**
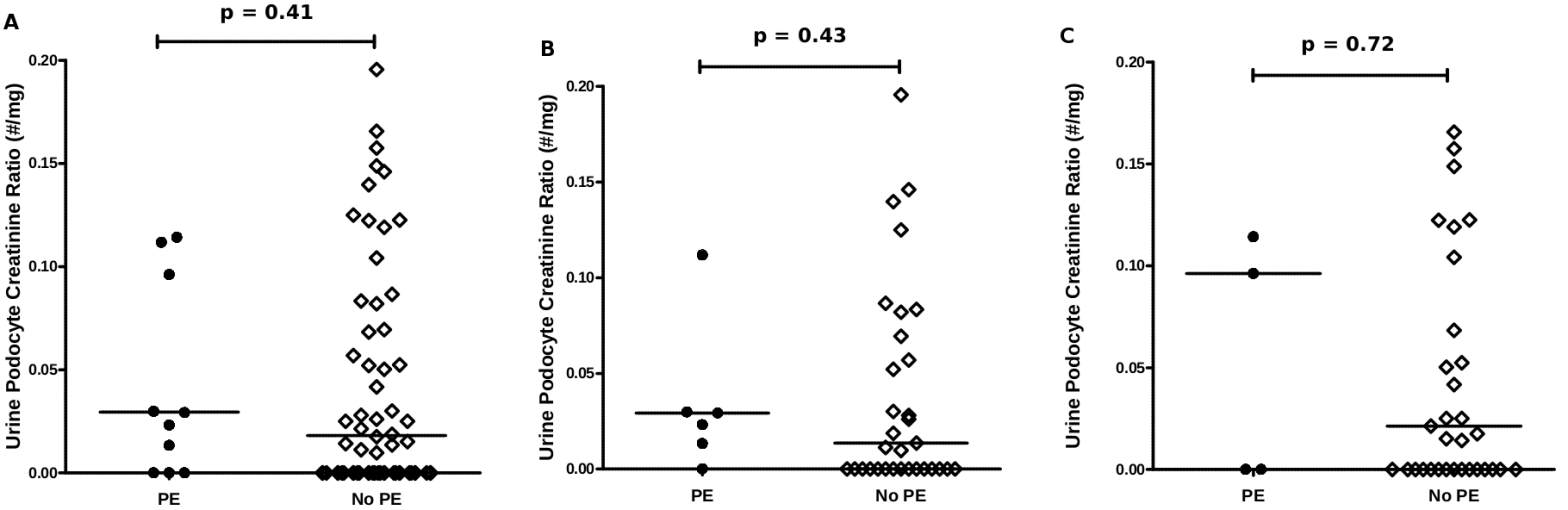
Dot plots of urine podocyte-to-creatinine ratios. A) Urine podocyte-to-creatinine ratio measurements in 2^nd^ and 3^rd^ trimester recruited patients who developed PE vs. patients who did not develop PE B) Urine podocyte-to-creatinine ratio measurements in 2^rd^ trimester recruited patients who developed PE vs. patients who did not develop PE C) Urine podocyte-to-creatinine ratio measurements in 3^rd^ trimester recruited patients who developed PE vs. patients who did not develop PE.

Images of podocyturia are shown in Figures 1A–1C, where Figure 1D demonstrates the negative control and Figure 1E represents the positive control. Measured pod/Cr is shown in Figures 2A–C. We did not observe significant differences in pod/Cr in patients who developed PE vs. those who did not (p = 0.41) (Figure 2A). When divided amongst trimesters, the differences remained statistically non-significant (p = 0.43 for 2^nd^ trimester and p = 0.71 for 3^rd^ trimester) (Figure 2B–C).

### Urinary albumin-to-creatinine ratio

**Figure 3:**
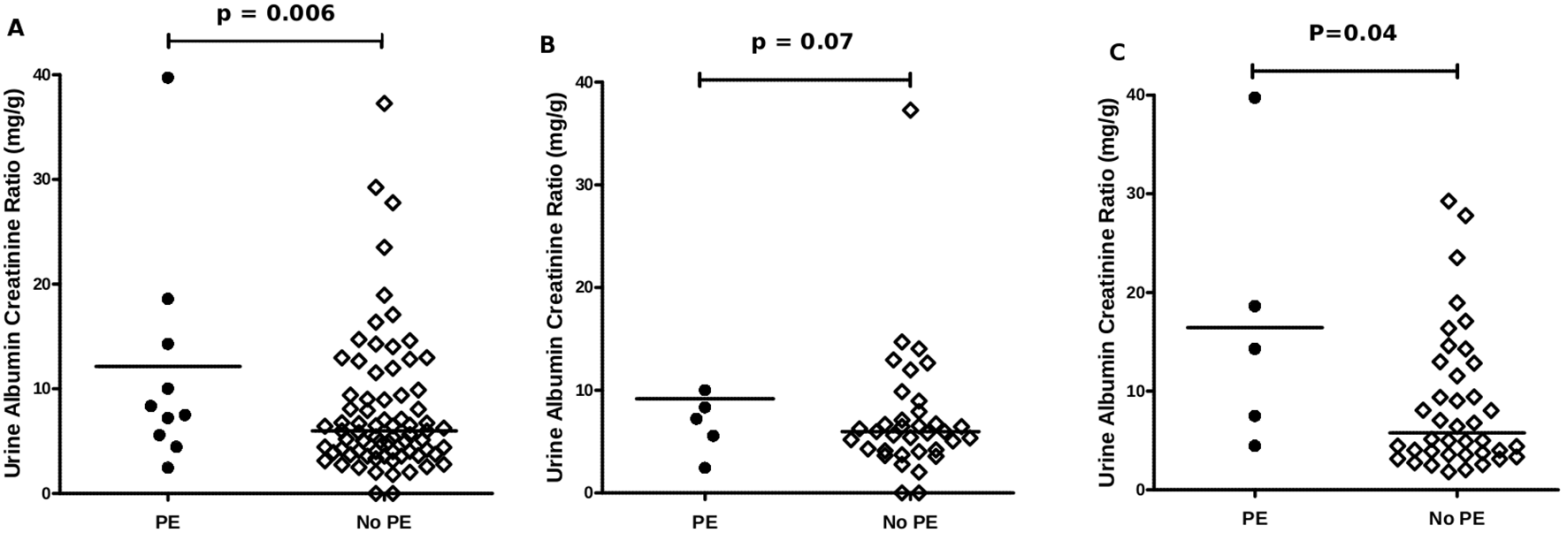
Dot plots of albumin-to-creatinine ratios. A) Urine albumin-to-creatinine ratio measurements in 2^nd^ and 3^rd^ trimester recruited patients who developed PE vs. patients who did not develop PE B) Urine albumin-to-creatinine ratio measurements in 2^rd^ trimester recruited patients who developed PE vs. patients who did not develop PE C) Urine albumin-to-creatinine ratio measurements in 3^rd^ trimester recruited patients who developed PE vs. patients who did not develop PE.

There was a significant difference on the alb/Cr between patients who developed PE from those who did not (p = 0.006) (Figure 3A). When divided amongst trimesters, the significance remained in the 3^rd^ but not 2^nd^ trimester (p = 0.04 vs. p = 0.07 respectively) (Figures 3B–C).

### Urinary nephrin-to-creatinine ratio

**Figure 4:**
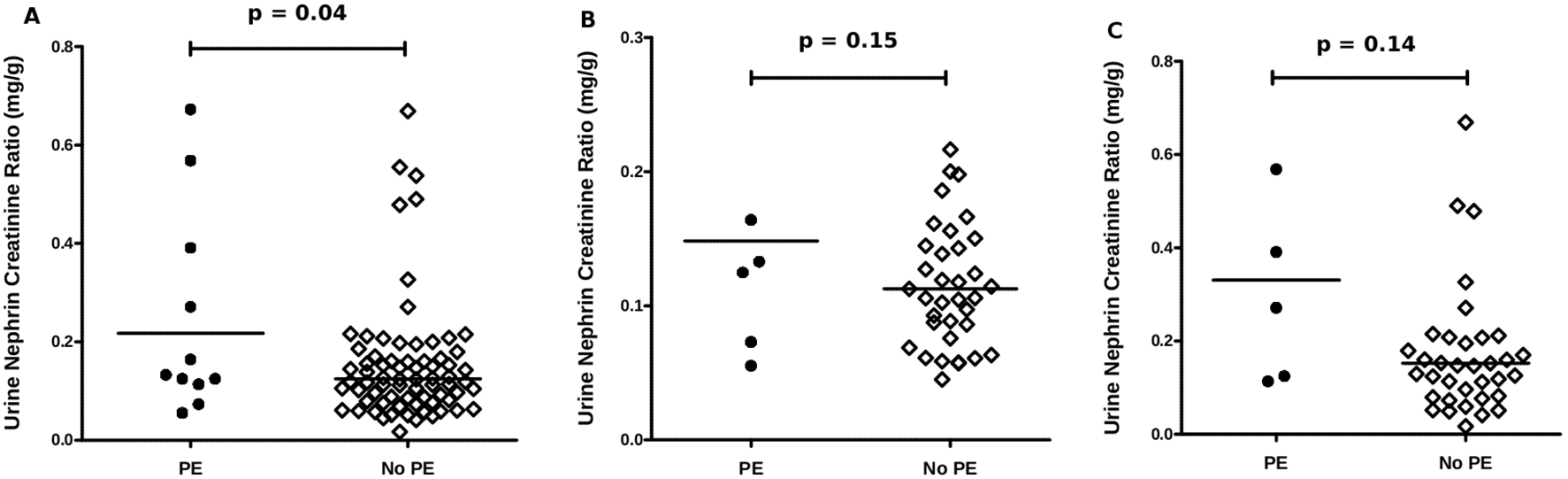
Dot plots of urine nephrin-to-creatinine ratios. A) Urine nephrin-to-creatinine ratio measurements in 2^nd^ and 3^rd^ trimester recruited patients who developed PE vs. patients who did not develop PE B) Urine nephrin-to-creatinine ratio measurements in 2^rd^ trimester recruited patients who developed PE vs. patients who did not develop PE C) Urine nephrin-to-creatinine ratio measurements in 3^rd^ trimester recruited patients who developed PE vs. patients who did not develop PE.

Measurements of the nephrin/Cr ratio revealed significant differences when the women who developed PE were compared to those who did not (p = 0.04) (Figure 4A). Subgroup analysis revealed that neither the 2^nd^ nor the 3^rd^ trimester recruited patients showed significant differences between any of the groups (p = 0.15 and p = 0.14 respectively) (Figure 4B–C).

### Test characteristics of all biomarkers

The test characteristics for all three biomarkers are listed in Table 2. The sensitivities proved to be the highest for pod/Cr at 70%, as compared with lower values for alb/Cr and nephrin/Cr (36% and 57% respectively). Only the alb/Cr ratio had a high specificity (96%). All three biomarkers exhibited poor positive predictive values (14–62%) but acceptable negative predictive values (89–91%). When analyzed amongst only the high risk individuals, the sensitivities of nephrin/Cr and pod/Cr improve to 86% and 79% respectively, while alb/Cr did not change. The positive and negative predictive values, however, do not change to any significant degree when examined only in high risk patients (Table 2B). Strong correlations were noted between alb/Cr and nephrin/Cr with urine protein-to-creatinine ratio (p<0.0001) (Table 3). Other significant correlations include alb/Cr and systolic BP (p = 0.0005), alb/Cr and diastolic BP (p = 0.01) as well as pod/Cr with systolic BP at delivery (p = 0.02). Interestingly, there was no significant correlation between pod/Cr with urine protein-creatinine ratio.

**Table 2:** Predictive characteristics of Markers Patients.

A: Predictive Characteristics of Markers in All Patients				
Biomarker	Sensitivity	Specificity	Positive Predictive Value	Negative Predictive Value
Urine albumin-to-creatinineratio (mg/g)	36%	96%	63%	89%
Urine nephrin-to-creatinineratio (mg/g)	57%	58%	19%	89%
Urine Podocyte-to-creatinineratio (#/mg)	70%	43%	14%	91%
**B: Predictive Characteristics of Markers in High Risk Patients**				
**Biomarker**	**Sensitivity**	**Specificity**	**Positive Predictive Value**	**Negative Predictive Value**
Urine albumin-to-creatinineratio (mg/g)	36%	94%	56%	87%
Urine nephrin-to-creatinineratio (mg/g)	86%	36%	23%	92%
Urine Podocyte-to-creatinineratio (#/mg)	79%	41%	22%	90%

**Table 3:** Correlation of Biomarkers with Clinical Parameters.

	1Urine Alb/Cr (mg/g)		2Urine Neph/Cr (mg/g)		3Urine Pod/Cr (#/g)	
	4 *R*	P value	*R*	P value	*R*	P value
Urine Protein-to-creatinine (g/g)	0.96	<0.0001	0.52	<0.0001	0.07	0.47
Delivery 5SBP	0.36	0.0005	0.16	0.12	0.25	0.021
Delivery 6DBP	0.26	0.012	0.15	0.14	0.17	0.10

1 Urine Alb/Cr: Urine albumin to creatinine ratio.

2 Urine Neph/Cr: Urine nephrin to creatinine ratio.

3 Urine Pod/Cr: Urine podocyte to creatinine ratio.

4 *R*: Spearman rho correlation.

5 SBP: systolic blood pressure.

6 DBP: diastolic blood pressure.

## Discussion

There has been substantial progress in research that focuses on the cause of PE (once dubbed “the disease of theories”). Though the exact causes have yet to be determined, identification of many possibly involved molecules has increased studies focusing both on prediction and verification of diagnosis [11]. For example, the circulating levels of pro- and anti-angiogenic proteins (e.g., sFlt-1, sEng, PlGF) have been linked to PE’s manifestations, including hypertension, proteinuria, and glomerular endotheliosis [16]. There are also data suggesting that podocyte associated protein expression is reduced, specifically synaptopodin and nephrin, while podocin expression remained unchanged [12]. This was accompanied by studies showing increases in the excretion of podocytes (podocyturia) using podocin as the marker which had a 100% sensitivity and specificity for the diagnosis of PE. We, on the other hand, were not able to reproduce these results using synaptopodin as our podocyte marker. In fact, in a study focusing on high risk patients, we observed a sensitivity and specificity of podocyturia to diagnose PE to be but 38% and 70% respectively [14]. More recently, Craici studying women during at the end of their second trimester described a 100% sensitivity using a podocin as a marker of podocyturia to predict PE [15]. Our results do not support such optimism as our sensitivities for all three markers do not appear to be high enough for clinical use.

The contrasting results between our findings and those reported by Craici et al might relate to methodologies and patient populations. We utilized the cytospin technique in which the urine samples are fixed immediately to avoid the errors that occur with cultivation of urinary cells. Growing urinary podocytes in cell culture is frequently limited by bacterial or fungal contamination. Furthermore, cells may proliferate, undergo apoptosis, or not attach to the culture dish, thereby falsely representing the true podocyte count [17]. Also, we identified podocytes by positive co-localization of podocin and synaptopodin to avoid false positive staining that may occur with the polyclonal antibody used to detect podocin. Craici et al reported using only the podocin antibody for staining. Furthermore, we included other high risk pregnant patients including DM, SLE, and CKD which may decrease the specificity of podocyturia as they are known to be present in non-pregnant individuals; the study by Craici et al, in contrast, examined podocyturia only in previously healthy women or those with gestational HTN.

As enticing as it may be to pursue a novel method to predict PE, the methodologic limitations of isolating podocytes in the urine persist. As mentioned above, there are practical concerns associated with isolating and identifying podocytes [17]. In addition, podocyte counting must be performed by an experienced cytopathologist, obviously impractical for a clinical test. Thus investigators have sought to avoid this highly technical procedure by measuring their urinary mRNA levels. Kelder and colleagues demonstrated that urinary mRNA levels were elevated in terms of nephrin, podocin in PE patients as compared with healthy pregnant women, the significance, interestingly, was lost amongst podocin mRNA levels between PE and gestational HTN [18]. This method is worth exploring as it has higher reproducibility, though it is also challenged by the inherent instability of RNA and the larger urine volume required for adequate RNA retrieval. Importantly, there also appears to be normal shedding of podocytes during pregnancy, as demonstrated by increased levels of intact podocytes [19] and urinary mRNA of podocin and nephrin in pregnant control patients as compared with non-pregnant controls [18]. This fact was re-iterated when human urinary podocytes were isolated in healthy patients [20]. We also detected podocyturia in healthy cohort. Hence, it appears that physiologic shedding of podocyte occurs both in the pregnant and non-pregnant population, and that this threshold must be quantified before we can use podocyturia to predict PE.

The strengths of this study are that it is prospective and includes a high risk patient population which may also present with podocyturia, such as DM and SLE [21], [22]. In order for a predictive marker to be validated, it needs to be studied in a confounding population. We also investigated alb/Cr and nephrin/Cr as comparison markers. The major limitation of this study is the sample size of the patients. However, our sample size is comparable to all other podocyturia studies we could locate to date. In fact, we have analyzed more samples for podocyturia in total (72 high risk and 13 low risk pregnant patients) when compared to other studies [13], [15], [19]. The non-significance of podocyturia patients who developed PE vs. those who did not may reflect insufficient statistical power. Nonetheless, the association of podocyturia with PE is not as strong as that for alb/Cr and nephrin/Cr. Thus, it appears unlikely that podocyturia would add additional information to existing clinical estimates of risk, and even more so if alb/Cr and nephrin/Cr were used.

## Conclusions

Podocyturia, nephrinuria, or albuminuria does not appear to be reliable markers to predict PE, though a “normal” alb/Cr may be useful to help rule out disease. We realize our notation of a relatively low sensitivity and specificity is not conclusive, but given the discrepancy of these observations to studies published prior to them, we suggest further studies are warranted. Currently, some underscore complex pathophysiology of the disease, suggesting different pathomechanisms especially between early and late PE, and suggest it is unlikely that any single test or cell type will be able to predict PE, while others disagree [23]. If the former view prevails, a panel of biomarkers reflective of this complexity may be ideal for diagnosis.
